# Advancements in prostate cancer segmentation: Integrating prostate zonal information

**DOI:** 10.1177/20552076261439004

**Published:** 2026-04-09

**Authors:** Aleksas Vaitulevičius, Jolita Bernatavičienė, Ieva Naruševičiūtė, Jurgita Markevičiutė, Mantas Trakymas, Povilas Treigys

**Affiliations:** 1 Faculty of Mathematics and Informatics, Institute of Data Science and Digital Technologies, 54694 Vilnius University, Vilnius, Lithuania; 2 277613 National Cancer Institute, Vilnius, Lithuania; 3 Faculty of Mathematics and Informatics, Institute of Applied Mathematics, 54694 Vilnius University, Vilnius, Lithuania

**Keywords:** mpMRI, artificial intelligence, prostate cancer segmentation, nnU-Net, ensembles, Dice Score Coefficient (DSC)

## Abstract

**Background and Objective:**

Prostate cancer is one of the most common cancers in men, and early diagnosis is critical. Segmentation of cancerous regions in multiparametric MRI is a key step. Deep neural networks, such as nnU-Net, perform well, and incorporating prostate zonal information may further improve accuracy.

**Methods:**

This study introduces four prostate cancer segmentation ensembles that integrate zonal data, compared with a baseline model, which uses zonal information as a separate input channel. Ensembles employ specific prostate zone cancer segmentation models trained with the nnU-Net method. To address variability in manual annotations, a new evaluation metric, the tolerant Dice Score Coefficient (*DSC*_*τ*_), is proposed, accounting for ground truth inaccuracies.

**Results:**

Ensemble 3 yields the best performance, with a 4.77% higher mean DSC and 6.17% higher mean *DSC*_*τ*_ than the baseline. Although the metrics of Ensemble 4 are slightly lower, it reduces false positives by 7.79% and uses fewer models (2 vs. 3), making it more efficient. Furthermore, the application of the Conover post hoc test for unreplicated blocked data shows that there is no statistically significant difference in performance metrics between the results of two ensembles. Thus, Ensemble 4 is the preferred approach for prostate cancer segmentation. Additionally, all ensembles achieve 5.03% to 7.13% higher mean *DSC*_*τ*_ values compared to the standard DSC, confirming the effectiveness of the new metric in handling segmentation uncertainties.

**Conclusion:**

The experiment results indicate that the proposed Ensemble 4 is the most suitable solution for the prostate cancer segmentation task. Moreover, the results also indicate that the proposed metric, *DSC*_*τ*_, accounts for ground truth segmentation errors.

## Introduction

The most recent cancer statistics, as presented in the article^1^ by Bray et al., indicate that among men worldwide, the incidence of prostate cancer is the second highest, and its mortality rate ranks fifth among all cancers. Considering the scale and severity of prostate cancer, an accurate segmentation or localization of cancerous regions is required to provide support for the Prostate Imaging Reporting and Data System (PI-RADS) assessment.

PI-RADS was introduced in the article^2^ by Barentsz et al. to help radiologists evaluate mpMRI for further diagnostic assessment. It is one of the most widely used techniques for standardized interpretation in prostate cancer diagnosis. This technique employs multiparametric (mp) magnetic resonance imaging (MRI). The latest PI-RADS version is 2.1, introduced in the paper^3^ by Turkbey et al. mpMRI consists of multiple sequences and the sequences used in PI-RADS v2.1 are T2-weighted (T2), Diffusion-Weighted Imaging (DWI), Apparent Diffusion Coefficient (ADC), and Dynamic Contrast-Enhanced (DCE) sequences. ADC and DWI are described in the article^4^ by Baliyan et al. DWI is acquired from an MRI scanner while ADC is derived from multiple DWI scans. Moreover, the T2 sequence is described in the article^5^ by Chavhan et al. Just as the DWI sequence, the T2 is acquired from an MRI scanner. All of these sequences are volumetric, anisotropic images. Lastly, the DCE sequence is described in the paper^6^ by Yankeelov and Gore, which presents the methodology of dynamic contrast-enhanced MRI based on repeated volumetric imaging over time after contrast agent injection to capture contrast uptake and washout kinetics. In this study, DCE is excluded because PI-RADS v2.1 places primary diagnostic weight on T2, ADC and DWI, with DCE having secondary and limited incremental value.

The assessment within PI-RADS v2.1 employs different mpMRI imaging sequences across prostate zones^3^ This is due to the fact that different imaging sequences are more effective in identifying cancerous regions within specific prostate zones. The PI-RADS v2.1 is assessed in the Transitional Zone (TZ) and the Peripheral Zone (PZ) of the prostate, as these zones account for the majority of the cancerous cases. The T2 sequence serves as the principal modality for evaluating TZ, while ADC and DWI are the primary sequences for assessing PZ. Thus, this paper proposes ensembles of deep neural network models trained on different mpMRI sequences to segment cancerous regions within specific prostate zones.

Prostate zone masks are employed for training the deep neural network models within the proposed ensembles, as well as for evaluating the performance of the ensembles. These masks are acquired by using separate models for the TZ and PZ segmentation (SM4TZPZS) workflow, which was proposed by Vaitulevičius et al. in the article.^7^ This workflow employs two deep neural network models: one dedicated to the segmentation of the TZ and the other to the segmentation of the PZ. The intersection of the segmentation outputs is assigned to the TZ. The prostate zonal mask is obtained by combining the outputs of the models into a single volume, which contains TZ and PZ. The paper^7^ contains the experiment results, which indicate that this workflow generalizes between different datasets better than the baseline workflow, single PZ and TZ segmentation model. Moreover, SM4TZPZS workflow has already been applied in previous work on prostate cancer localization, specifically in the study^8^ by Roman et al. In addition, several post-processing steps are introduced in this study in order to correct the result of the SM4TZPZS workflow.

In total four cancerous region segmentation ensembles are proposed in this paper. The first two ensembles employ distinct deep neural network models, each trained to segment cancerous regions in the specific prostate zone. The models of the first ensemble adhere strictly to the PI-RADS v2.1 assessment guidelines, utilizing only the principal sequences specific to each zone. In contrast, the models of the second ensemble incorporate all available sequences for both prostate zones. The third ensemble integrates the baseline model together with the second ensemble. The final ensemble utilizes the baseline model and the model which employs all sequences to segment cancerous regions exclusively within the PZ. This paper presents the experiment in which the proposed ensembles are compared with the baseline model, which uses prostate zone information as a separate channel.

Moreover, all deep neural network models used in the ensembles as well as the baseline model are trained with the state-of-the-art method for medical segmentation, no-new U-Net (nnU-Net). This method was introduced by Isensee et al.^9^ nnU-Net and its variations were widely employed in various medical segmentation experiments such as the ones presented in articles.^7,10–14^ In addition, the research presented in this paper employs the extended nnU-Net method incorporating a preprocessing step introduced by Jucevičius et al.^14^ This step involves converting anisotropic volumetric MRI data into isotropic. The experiments presented by Jucevičius et al. indicate that this step improves the accuracy of the nnU-Net method.

Lastly, a new evaluation metric, the tolerant Dice Score Coefficient (*DSC*_*τ*_), is proposed in this paper. The experiment results provided in the article^15^ by Chen et al. indicate that prostate cancer segmentation is a very difficult task, and the results of segmentation are highly variable even among experienced radiologists. Therefore, the proposed metric accounts for segmentation errors of the ground truth that may occur as a result of the prostate cancer segmentation process.

Overall, in this paper:

• Four ensembles are proposed. The proposed ensembles are compared with the baseline model, a model that uses prostate zone information as a separate channel. The results of the experiment presented in this paper show that the most suitable solution to the prostate cancer segmentation task is the ensemble comprised of the baseline model and the model that employs all sequences to segment cancerous regions exclusively within the PZ.
• A novel evaluation metric is proposed, which is an extended DSC that considers the ground truth’s segmentation error. In addition to the original DSC, the results of the experiment described in this paper are evaluated using the proposed metric. The results of this experiment affirm that *DSC*_*τ*_ accounts for ground truth segmentation errors, as the means obtained from the collected *DSC*_*τ*_ are higher than those from the original DSC.

The rest of this paper is structured as follows: Related works section reviews related work, Methods section provides a detailed description of the ensembles, Experiment section describes the dataset used in the experiment and experiment methodology and the implementation details, Results section presents the experimental results, Discussion section discusses the results and Conclusion section concludes the paper.

## Related works

**Figure 1.**
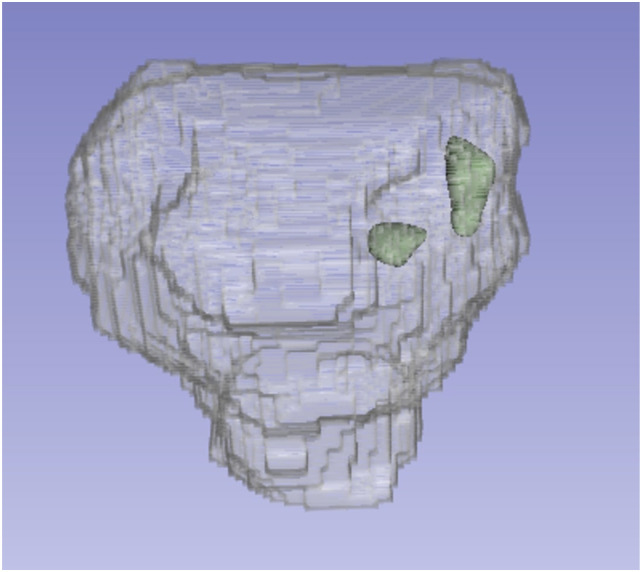
The example of the ground truth of prostate cancer segmentation task. The green volume is the cancerous region. The transparent region is the prostate mask.

**Figure 2.**
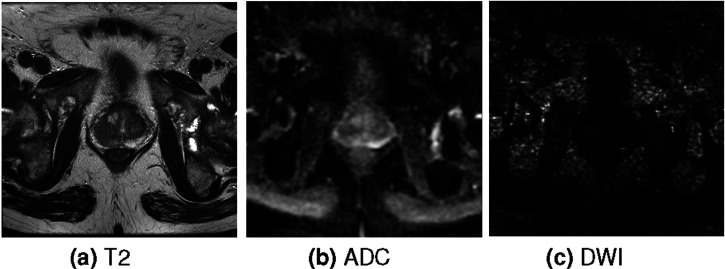
The example of a single slice of each sequence.

This section reviews works employing deep neural networks and prostate zone information for prostate cancer segmentation tasks. The example of the ground truth of this task is displayed in Figure 1 where the green volume is the cancerous region and the transparent region is the prostate mask. The segmentation is performed using mpMRI sequences T2, ADC and DWI. The examples of these sequences are shown in Figure 2.

A lot of research has already been performed on employing prostate zone information and deep neural networks for prostate cancer segmentation. One of it is published by Hosseinzadeh et al. in the papers.^16,17^ In this research, the authors demonstrated the advantages of incorporating probabilistic prostate zonal segmentation within a 2D computer-aided detection system. Their findings published in the article^16^ indicate that including PZ and TZ segmentations leads to an average increase of 5.3% in detection sensitivity and between 0.5 and 2.0 false positives per patient. Furthermore, the findings presented in the paper^17^ indicate that training models with training sets of different sizes draw the same conclusion, prostate zone masks improve the accuracy of prostate cancer segmentation.

The other research whose proposed solution uses an attention mechanism is provided in the paper.^18^ The authors of this paper propose a workflow as a solution. This workflow uses a U-Net variation, Dual-Attention U-Net. Moreover, the research also contains the comparison of their proposed workflow with nnU-Net, Attention U-Net, and Dual-Attention U-Net architectures. The comparison indicates that their proposed workflow achieves the best results. Meanwhile, Dual-Attention U-Net achieves slightly better results than nnU-Net on one of their test sets and worse on the other. Attention U-Net achieves worse results than nnU-Net and Dual-Attention U-Net architectures on both test sets.

More research is provided in the article^19^ by Duran et al. Their research examined the impact of employing a whole prostate mask and a single prostate zone mask for segmentation models. The use of PZ achieved 1.8% higher sensitivity and 1.4 fewer false positives per patient than the use of the whole prostate mask. However, employing a TZ mask for the segmentation model resulted in 13.9% lower sensitivity and 1.4 lower false positive per patient than employing the entire prostate mask. The authors attribute the inaccurate performance of the TZ mask to a low number of lesions in the TZ. Moreover, according to the articles^20^ by Oto et al.,^21^ by Rosenkrantz et al. and^22^ by Yu et al. cancerous regions in the TZ are difficult to distinguish from benign hyperplasia nodules. The authors of the paper^21^ also indicate that the diffuse background heterogeneity and multinodularity of the TZ complicate the cancerous region segmentation of this zone. Meanwhile, the authors of the article^22^ indicate that stromal asymmetry may mimic a cancerous region within TZ, whereas chronic prostatitis may produce similar appearances within the PZ. Lastly, the research presented in the paper^23^ by Yu et al. indicates that cancerous regions appear less often in TZ than in PZ. Therefore, one of the ensembles proposed in this study employs a PZ mask to a greater extent than the TZ mask.

Overall, a plethora of papers contain research that uses prostate zone information in deep neural network models as a channel-wise input. Examples include the research provided by Saha et al. in the article,^18^ the research published by Zheng et al. in the article^24^ and the research described in the paper^25^ by Mehta et al. Thus, this paper focuses on exploring other possible incorporations of prostate zone information into deep neural network models as a solution to a prostate cancer segmentation task.

Lastly, the results of prostate cancer segmentation have a high variance among even experienced radiologists, as indicated by the results of the experiment provided in the article.^15^ The experiment included 4 radiologists who were tasked with segmenting prostate cancer within a single slice of the anterior zone and the PZ. Their segmentations were compared by calculating the mean DSC. The resulting mean DSC for the segmentations within PZ is 0.81, while for the segmentations within the anterior zone is 0.58. Therefore, the metric proposed in this study addresses this issue.

## Methods

### Compared ensembles

In this study, four ensembles are introduced. The result of the ensemble is a disjunction of the outputs of all deep neural network models used in the ensemble. As mentioned before, these models are obtained using the nnU-Net method with an additional preprocessing step proposed by Jucevičius et al.^14^ The primary distinction between these models lies in the input of the model. Each model uses different sequences as input, and each used sequence is a separate channel of the input. Furthermore, a different mask is applied to the input of each model by setting voxels that do not overlap with the mask to zero. This masking strategy is an integral component of the nnU-Net framework and is therefore selected over alternative approaches, such as soft masking. Thus, the following models are used in the proposed ensembles:

• *TZ* (*T*2) - this model uses only the T2 sequence as an input to segment cancerous region within TZ.
• *PZ* (*ADC*, *DWI*) - this model uses the ADC and DWI sequences as an input to segment cancerous region within PZ.
• *TZ* (*All*) - this model uses all three sequences as an input to segment cancerous region within TZ.
• *PZ* (*All*) - this model uses all three sequences as an input to segment cancerous region within PZ.
• *prostate* (*All*, *Zones*) - this model uses all three sequences to segment cancerous region within the whole prostate. The prostate masks used for the experiment are disjunctions of TZ and PZ masks. Furthermore, the state-of-the-art method to employ prostate zone information in prostate cancer segmentation tasks is to use prostate zone masks in deep neural network models as a separate input channel together with other mpMRI sequences. This method was employed in the experiments presented in papers.^16,18,25^ Therefore, the prostate zone masks and mpMRI sequences are used as a channel-wise input of the *prostate* (*All*, *Zones*) model. This model is the baseline model used in the comparison.

**Figure 3.**
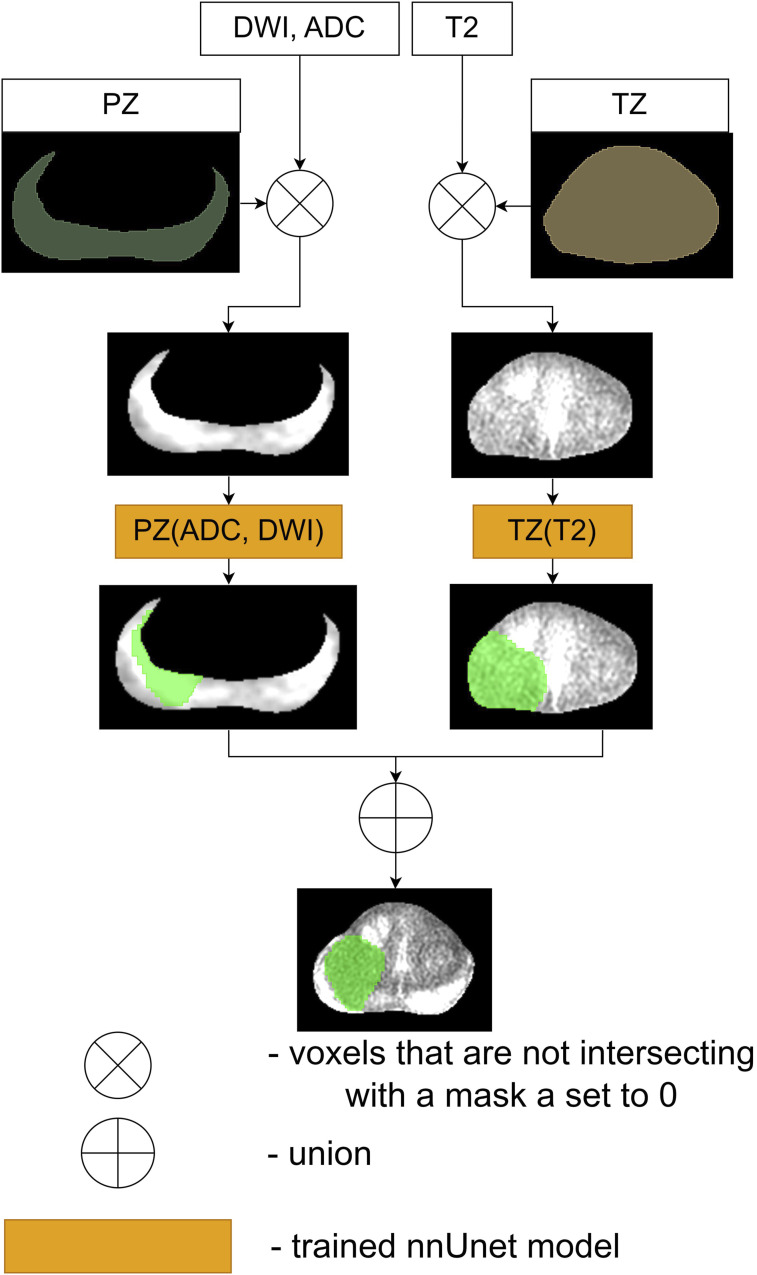
The workflow of Ensemble 1.

**Figure 4.**
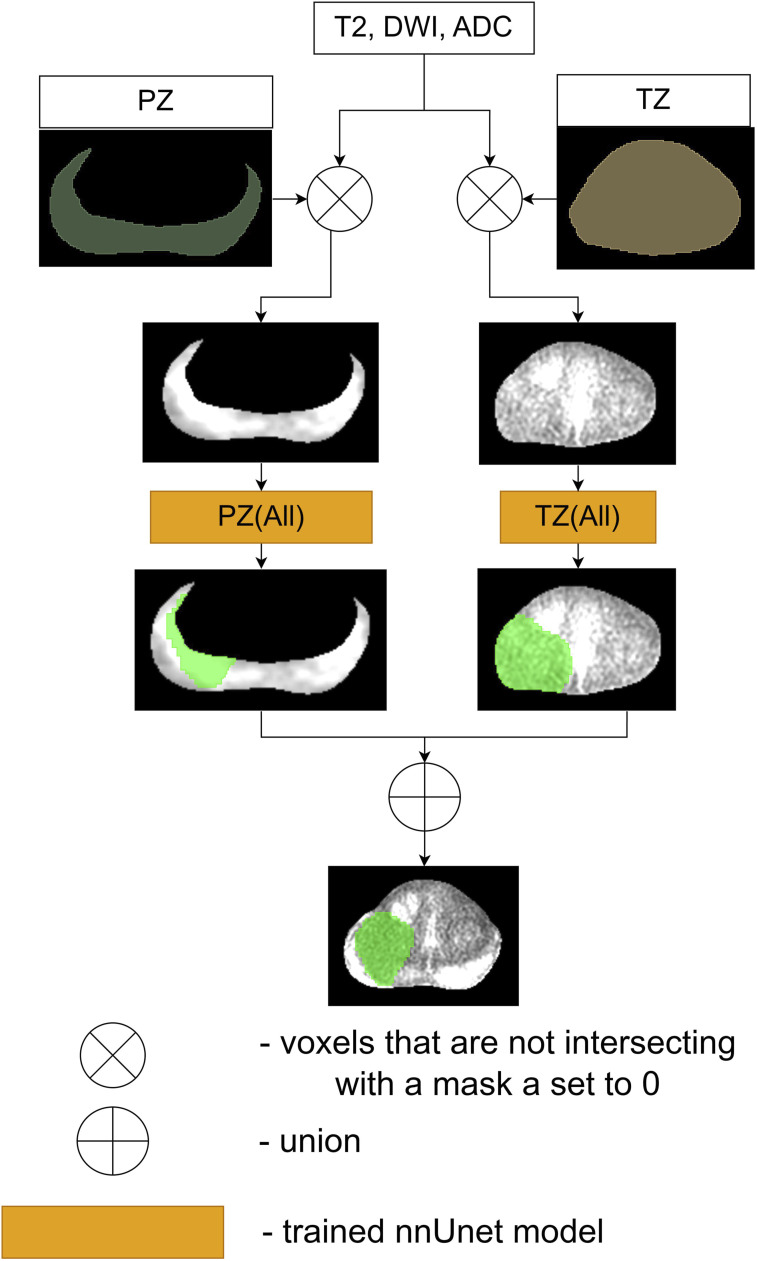
The workflow of Ensemble 2.

**Figure 5.**
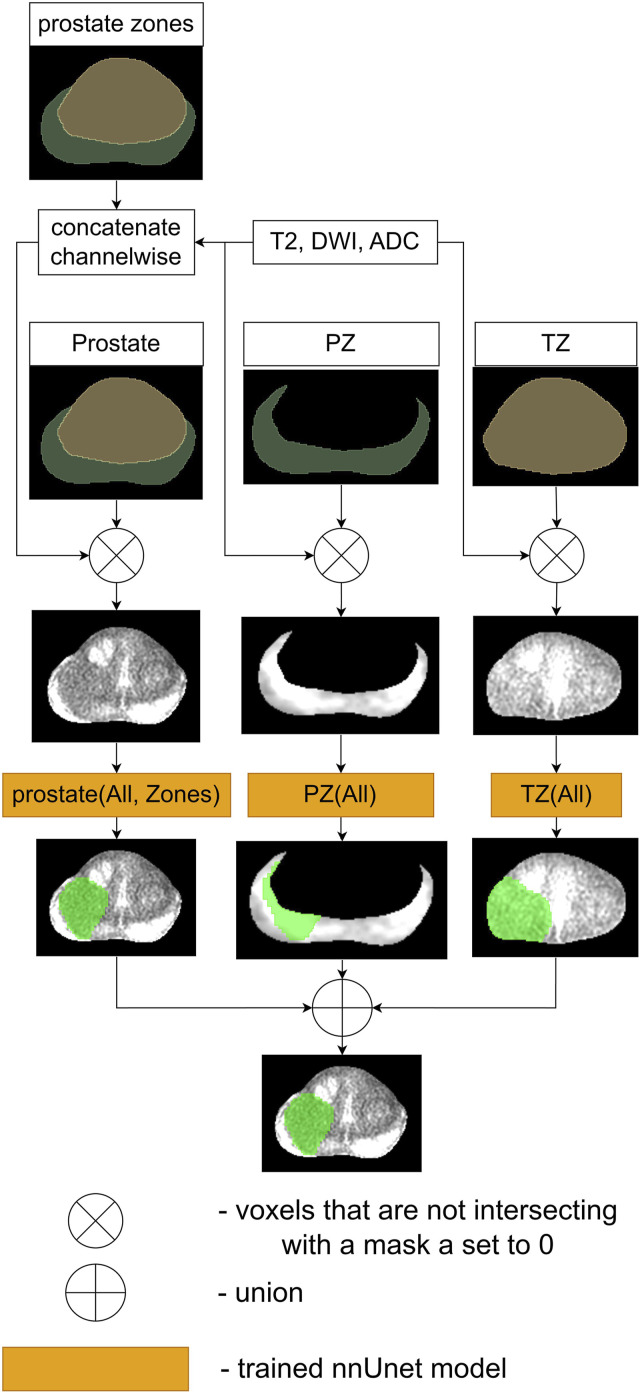
The workflow of Ensemble 3.

**Figure 6.**
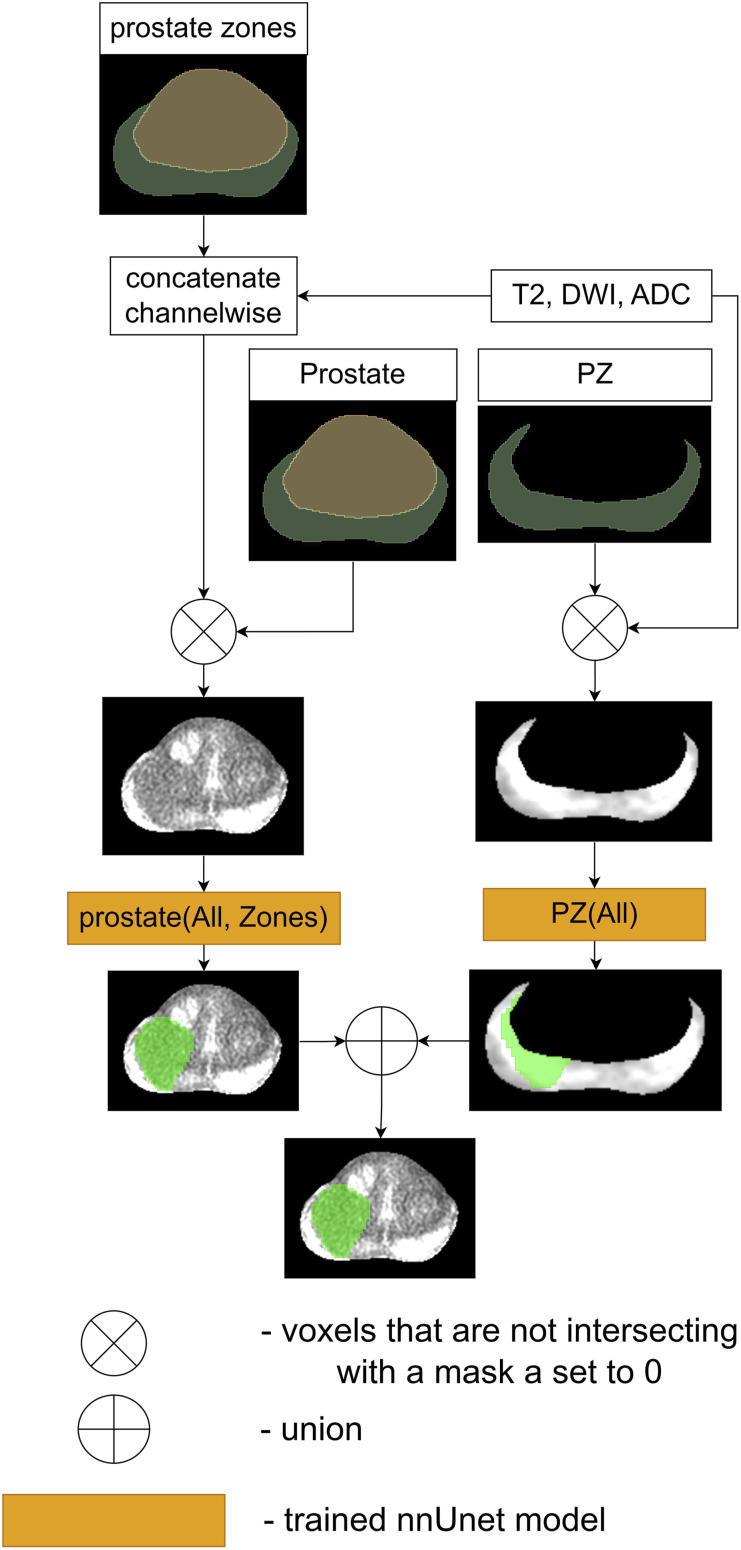
The workflow of Ensemble 4.

The first proposed and compared ensemble, denoted as Ensemble 1, is visualized in Figure 3. Green areas in Figures 3–6 represent the result of cancerous region segmentation. As mentioned in the introduction, the principal sequence for evaluating prostate cancer in TZ is T2. Meanwhile, the principal sequences for evaluating prostate cancer in PZ are ADC and DWI. Therefore, this ensemble is the disjunction of *TZ* (*T*2) model’s result and *PZ* (*ADC*, *DWI*) model’s result. Formally - *Ensemble*1 = *TZ* (*T*2) ∨ *PZ* (*ADC*, *DWI*).

The schematic of the second proposed ensemble, denoted as Ensemble 2, is provided in Figure 4 While each prostate zone has its own principal sequences, radiologists typically employ all three sequences to assess cancerous regions in both zones. Therefore, this ensemble is the disjunction of the results of the models *TZ* (*All*) and *PZ* (*All*). Considering that the input of the model *TZ* (*All*) is three times bigger than *TZ* (*T*2) and the input of the model *PZ* (*All*) is 1/3 times bigger than *PZ* (*ADC*, *DWI*), Ensemble 2 is slower than Ensemble 1 during the inference and the training processes. Therefore, if Ensemble 1 and Ensemble 2 achieve similar results, then Ensemble 1 is preferred over Ensemble 2. The equation of Ensemble 2 is *Ensemble*2 = *TZ* (*All*) ∨ *PZ* (*All*).

The third proposed ensemble, denoted as Ensemble 3, is provided in Figure 5. Firstly, the baseline model, *prostate* (*All*, *Zones*), is used to create this ensemble. Furthermore, it is more likely that Ensemble 3 will achieve better results than Ensemble 2 as its input contains more information. Therefore, this ensemble is the disjunction of the *TZ* (*All*) model’s result, *PZ* (*All*) model’s result and *prostate* (*All*, *Zones*) model’s result. Overall, the accurate performance of this ensemble would indicate that *prostate* (*All*, *Zones*) model and ensemble 2 are complementary to each other. The equation of this ensemble is *Ensemble*3 = *TZ* (*All*) ∨ *PZ* (*All*) ∨ *prostate* (*All*, *Zones*).

The schematic of the last proposed ensemble, denoted as Ensemble 4, is visualized in Figure 6. Yu et al. in the paper^23^ provide a summary of the epidemiological literature. This summary contains the distribution of prostate cancer instances in prostate zones. Approximately 20% of instances appear in TZ. This means that the data is imbalanced, and prostate cancer in TZ may not have enough instances for the deep neural network model training. Moreover, the research in the articles^20–22^ indicates that detecting prostate cancer in TZ is generally more complicated than in PZ.

Therefore, this ensemble is the disjunction of the results of the models *PZ* (*All*) and *prostate* (*All*, *Zones*). Overall, if this ensemble in the experiment bears performance just as accurate as Ensemble 3, then it will indicate that the *TZ* (*All*) model does not significantly influence the result of Ensemble 3. Formally - *Ensemble*4 = *PZ* (*All*) ∨ *prostate* (*All*, *Zones*).

### Performance evaluation

Accurate evaluation of prostate cancer segmentation is inherently challenging due to the substantial inter observer variability in delineating tumour boundaries, even among expert radiologists and pathologists. This variability arises from indistinct lesion margins, heterogeneous tumour appearance on mpMRI, and partial volume effects, leading to small but clinically insignificant boundary discrepancies that are nevertheless penalized heavily by conventional overlap based metrics such as the Dice Score Coefficient (DSC). Thus, in this research, a new metric, the tolerant Dice Score Coefficient, is proposed. *DSC*_*τ*_ is an extended DSC^26^ that considers a segmentation error of the ground truth which may arise due to the nature of prostate cancer segmentation. The original DSC is incorporated into the loss function of the nnU-net method. Moreover, DSC has been widely used in prostate MRI segmentation studies, particularly for whole gland and zonal prostate segmentation. This metric is used in experiments, such as those presented in the articles.^27–29^ Furthermore, DSC has also been used in lesion segmentation works to assess spatial overlap, such as.^18,25,30^ The equation for calculating the original DSC is provided in Equation (1). In this equation, *TP* is a volume of the predicted cancerous region that overlaps with the ground truth; *FN* is a volume of the ground truth annotation which does not overlap with the prediction, and *FP* is a volume of the prediction that does not overlap with the ground truth. The volumes *TP*, *FP* and, *FN* are calculated as numbers of voxels.

(1) $$DSC=\frac{2TP}{2TP+FP+FN}.$$

Metrics such as area under the receiver operating curve and average precision, as employed in the PI-CAI challenge,^31^ and in the research published in the article,^18^ are well suited for lesion level detection and case level clinical decision support. In contrast, this study focuses on voxel wise segmentation of prostate cancer lesions, where spatial overlap and boundary accuracy are of primary interest. Therefore, DSC was selected as the principal evaluation metric.

**Figure 7.**
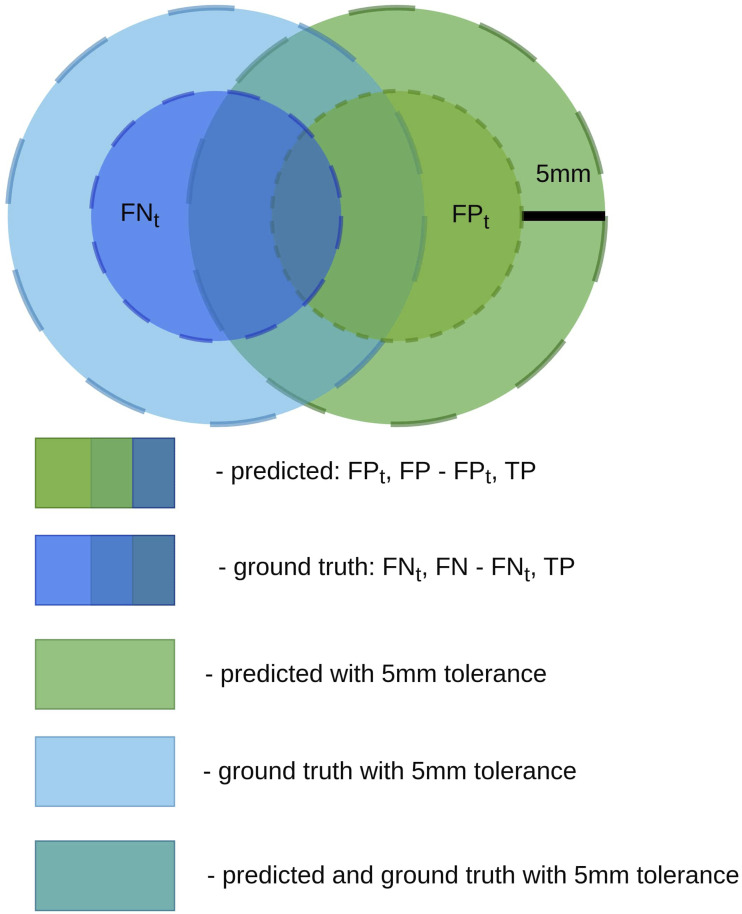
Venn diagrams of *FN*_*τ*_ and *FP*_*τ*_.

The proposed metric, *DSC*_*τ*_, relaxes the penalization of false positives and false negatives within a predefined spatial margin while preserving the definition of true positives. This design reflects the clinical reality that minor boundary deviations are often within the range of annotation uncertainty and should not be interpreted as segmentation failure. The proposed modification of DSC is Equation (2) where *FN*_*τ*_ is the number of tolerant *FN* and *FP*_*τ*_ is the tolerant *FP*. Tolerant *FN* and tolerant *FP* are metrics which consider segmentation errors of the ground truth. *FN*_*τ*_ is calculated by dilating the predicted volume, while *FP*_*τ*_ is calculated by dilating the ground truth volume. The dilation operation is described in the paper.^32^ This operation is a morphological operation that expands foreground regions in a binary image by adding pixels to object boundaries. The dilation operation is performed by sliding a structuring element over the image and replacing each pixel with the maximum value within the neighbourhood defined by the structuring element. This operation is commonly used to fill small gaps, connect nearby structures, and enhance object boundaries. Both dilations are performed with an annulus-shaped kernel. The radius of the kernel is adjusted so that the result of the dilation makes the dilated regions wider by a fixed length, called tolerance. Custom metrics were also used in the experiments, described in the papers.^24,33^ The metric used in the paper^33^ expands the prostate cancer segmentation by 5 mm. Furthermore, the authors of the paper^24^ consider predicted local maxima of prostate cancer probability maps as true positives if they are located within 5 mm of any prostate cancer region. Thus, the chosen tolerance for the experiment of this paper is 5 mm. The Venn diagram which illustrates the calculation of *FN*_*τ*_ and *FP*_*τ*_ is presented in Figure 7.

(2) $$DSC_{\tau }=\frac{2TP}{2TP+FN_{\tau }+FP_{\tau }}$$

Similar tolerant Dice Score Coefficient metrics have been introduced in the past. One of such metrics have been proposed in the paper.^34^ However, unlike *DSC*_*τ*_, proposed in this study, the tolerant Dice Score Coefficient, proposed by the authors of the article,^34^ contains *TP* calculated with tolerance. Thus, the novel metric, *DSC*_*τ*_, proposed in this paper, maintains the strict definition of correctly identified tumour regions, thereby preserving sensitivity to meaningful lesion detection while reducing undue penalization of ambiguous boundary regions. Another metrics similar to *DSC*_*τ*_ are surface DSC, proposed in the article,^35^ weighted DSC (WDC), proposed in the study,^36^ and Added Path Length (APL), proposed in the paper.^37^ These metrics differ significantly from *DSC*_*τ*_, introduced in this study. Surface DSC and APL assess the overlap of two surfaces rather than volumetric overlap as is done by the conventional DSC and *DSC*_*τ*_. Lastly, unlike the *DSC*_*τ*_, WDC takes into account the distance between the predicted and ground truth voxels in calculating volumetric overlap. It mitigates the impact of boundary discrepancies relative to the conventional DSC, but still penalizes them more than the *DSC*_*τ*_.

In this paper, the results of the experiment are evaluated with the original DSC and the proposed *DSC*_*τ*_.

Additionally, Friedman tests^38^ are employed to evaluate statistical significance between related groups, specifically comparing ensembles and the baseline model. This non-parametric test is suitable for analysing two or more samples, particularly when the data exhibit non-normal distributions or involve small sample sizes. The Friedman test determines whether significant differences exist in the mean ranks of the groups, making it well-suited for repeated measures designs.

Finally, the lesion level metrics are reported in this study. These include the distributions of produced lesion counts for each compared ensemble and the baseline model, as well as the distributions of missed lesions, which correspond to lesion level false negatives. In addition, the distributions of spurious foci predicted by each ensemble and the baseline model are reported, representing lesion level false positives.

## Experiment

### Data

The dataset used in the experiment is provided by the Lithuanian National Cancer Institute (NVI) under the terms of the bioethical agreement (number 2020/5-1229-714). It consists of mpMRI data, which consist of T2, DWI and ADC sequences. This dataset was collected from 11 Lithuanian centres. The original cohort consists of 146 patients. However, 26 patients are excluded due to the noise such as a hip implant or missing sequences, resulting in the dataset of 120 patients. Each centre employed a different MRI scanners with different parameters. Seven of these centres used MRI scanners with a magnetic field strength of 1.5T, while the remaining centres employed scanners with the strength of 3T. Moreover, the mpMRI scanners used different echo time (TE) and repetition time (TR) when scanning each case. The statistics of these parameters are provided in Table 1 where Seq column represents the sequence (T2 or DWI) and Param column indicates the parameter (TE or TR). The reported statistics consist of minimum, maximum, mean and median used TE and RT. All of the used scanners produced volumetric images with voxel spacing between slices of 2.5 mm. Meanwhile, an in-slice voxel spacing ranges between 0.39 to 0.78 mm.

**Table 1.** Statistics of echo time (TE) and repetition time (TR) for the acquired T2 and DWI sequences (milliseconds).

Seq	Param	Min	Mean	Median	Max
T2	TR	3000	5334.74	4310.31	9847
	TE	85	132.81	120	162
DWI	TR	1294.29	4114.89	3918.35	10267
	TE	66.19	79.57	78.25	93.8

**Figure 8.**
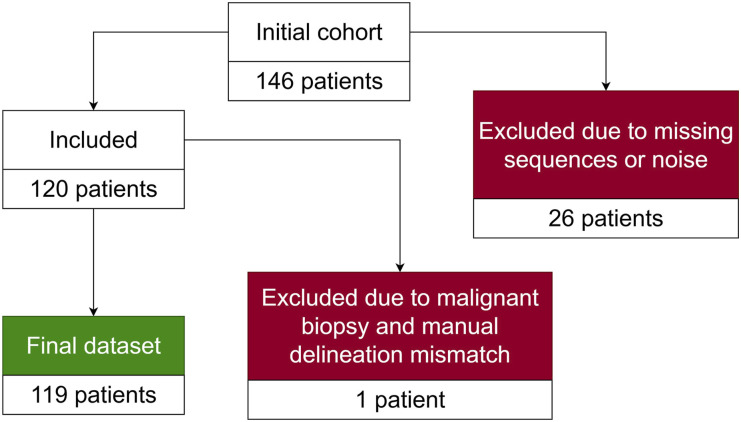
Patient selection flow diagram.

Moreover, the dataset includes prostate, cancerous region and biopsy masks. The cancerous region masks were manually delineated by a single radiologist of 10 years experience. The annotation process was completed prior to the experimental procedures. Thus, the annotator was blinded to the output of the compared ensembles and the baseline model. The biopsies were performed by the interventional radiologist with 15 years of experience. One patient had manually delineated cancerous regions that was not confirmed by a biopsy assigned a malignant diagnostic category, despite having other biopsies classified as malignant. Thus, this patient is excluded from the remaining cohort, resulting in the final dataset with the size of 119 cases. The complete flow diagram of patient selection is provided in Figure 8. The youngest patient in the final dataset is 46 years old, while the oldest one is 82. The average and median age of patients is 64 years old. On average, 15 biopsies were taken per patient, while the median number is 16. The highest number of biopsies performed on a patient is 25, while the lowest is 3. The distribution of the biopsy results by Gleason scores (GS) are reported in Table 2, where GS column indicates the Gleason Score and Category column indicates the corresponding diagnostic category. The No of patients column represents the number of patients who contain at least a single biopsy of the given GS, No of biopsies column indicates the total number of biopsies with that Gleason score and biopsies/patient represents the mean number of biopsies with the given Gleason score among patients with at least one such biopsy. Furthermore, the dataset is expanded with prostate zone masks. All sequences and masks are volumetric.

**Table 2.** Gleason score distribution.

Category	GS	No of patients	No of biopsies	Biopsies/Patient
Benign	0+0	112	1316	12
Undetermined	3+3	72	293	4
Malignant	3+4	34	97	3
Malignant	4+3	16	31	2
Malignant	4+4	5	11	2

**Figure 9.**
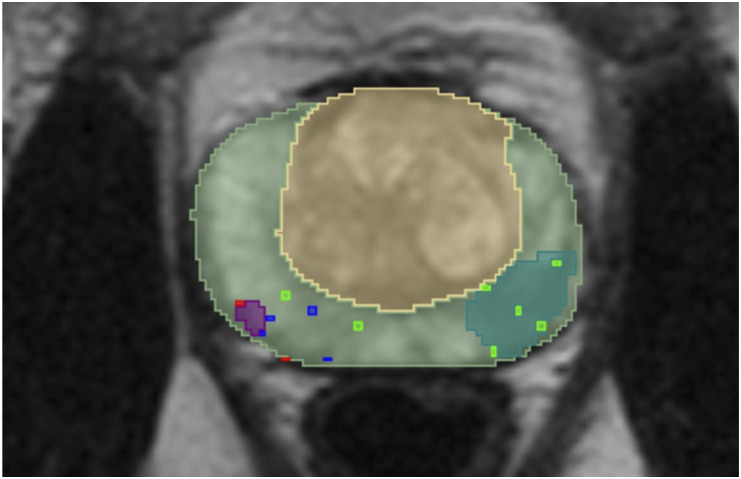
The example of a single slice, where one manually delineated lesion intersects with a malignant biopsy mask while the other does not.

**Figure 10.**
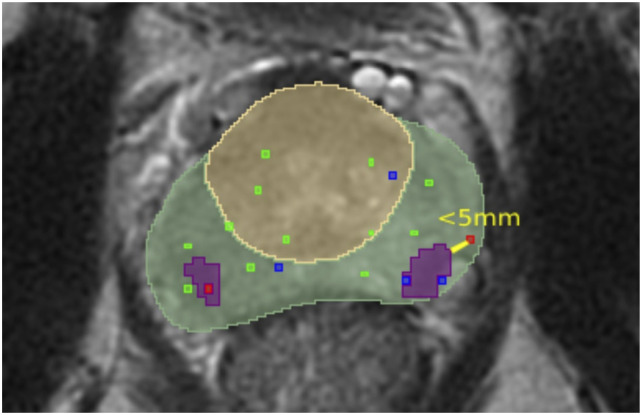
The example of a single slice, in which one manually delineated lesion is closer than 5 mm from the malignant biopsy.

The ground truth is acquired by first calculating the connected components within manual segmentations. Each component corresponds to a single manually delineated lesion within the segmentation. The ground truth is considered manually delineated lesions confirmed by a malignant biopsy. The lesion is considered confirmed by a malignant biopsy if it satisfies one of the two conditions. The examples of those conditions are illustrated in Figures 9 and 10 where the light green area is a PZ, the yellow area is a TZ, green, blue, and red dots represent biopsies with benign, undetermined, malignant results respectively and the purple areas represent the manually delineated lesions which are considered as a ground truth while the areas coloured in the light blue/green colour are lesions which are not included in the ground truth. Those conditions are:

• The manually delineated lesion intersects with a malignant biopsy mask. The example of a single slice, in which one lesion (purple area) satisfies this condition, is presented in Figure 9.
• Malignant biopsy mask is located within a 5 mm radius around the manually delineated lesion. According to the articles^39^ by Sahni et al. and^40^ by Bhat et al. the prostatic biopsies are performed using a standard grid. The grid contains evenly spaced holes with a 5 mm distance. Hence, this spacing can introduce an error of a 5 mm radius. The example of a single slice, in which one lesion (purple area) satisfies this condition and another satisfies the first condition, is presented in Figure 10.

The example of a single slice, in which one manually delineated lesion (area coloured in light blue/green) does not satisfy any of the conditions, is presented in Figure 9. The resulting distribution of lesions across prostate zones within the final dataset is provided in Table 3. In this table No of lesions column indicates the lesion count, Prostate column indicates the number of whole prostate glands containing the corresponding number of lesions, TZ and PZ columns indicate the number of transitional zones and peripheral zones, respectively, containing the given number of lesions. The lesion size statistics across prostate zones are reported in Table 4, where Zone column indicates prostate zone. These statistics consist of minimum, maximum, mean and median lesion sizes (*mm*^3^).

**Table 3.** The distribution of cancerous lesions across prostate zones.

No of lesions	Prostate	TZ	PZ
2	6	2	6
1	36	32	35

**Table 4.** Lesion size statistics. The sizes are reported in *mm*^3^.

Zone	Min size	Mean size	Median size	Max size
Prostate	71.38	1557.71	905.53	7694.17
TZ	0.67	1037.24	440.89	7283.42
PZ	12.98	670.9	412.5	3356.48

**Figure 11.**
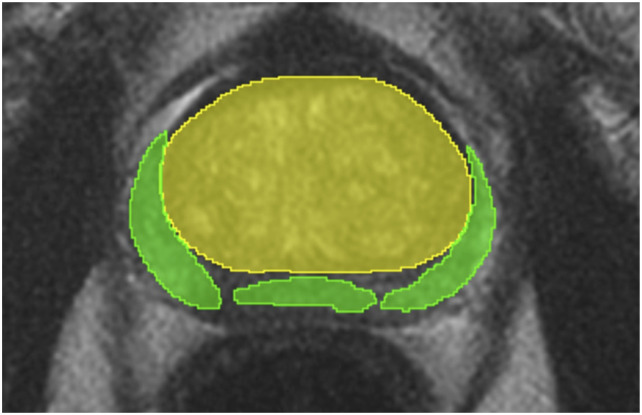
A single slice of unprocessed SM4TZPZS workflow result where green area represents PZ and yellow area represents TZ.

The prostate zone masks are acquired using the SM4TZPZS workflow, which was proposed by Vaitulevičius et al. in the article.^7^ However, the acquired prostate zones have faults – there are gaps between the zones, the zones overlap. These faults are caused by limitations of the deep neural network models, such as prediction uncertainty at zone boundaries and partial volume effects. As a result, these errors can negatively affect the performance of compared ensembles and the baseline model. An example of such faults is presented in Figure 11 where the yellow area represents a TZ and the green area represents a PZ. Therefore, a further post-processing of the workflow result is designed in this study. The post-processed PZ is acquired using a function (3). Meanwhile, a post-processed TZ is acquired using a function (4).

(3) $$\begin{array}{l} f_{PZ}\left(PZ,TZ\right)=dilate\left(PZ\right)\wedge closing\left(PZ\vee TZ\right)\wedge \\ \wedge \neg TZ \end{array}$$

(4) $$\begin{array}{l} f_{TZ}\left(PZ,TZ\right)=dilate\left(TZ\right)\wedge closing\left(PZ\vee TZ\right)\wedge \\ \wedge \neg f_{PZ}\left(PZ,TZ\right) \end{array}$$

In these functions, *dilate* is a dilation operation with a ball kernel of 5-pixel radius. Furthermore, *closing* is a binary morphological closing operation, which is defined as a dilation operation followed by an erosion operation. The erosion operation was introduced in the paper^32^ as well as the dilation operation. The dilation of PZ mask in the function (3) fills small gaps that occur between PZ and TZ regions. Next, the conjunction with the closing of the union of the unprocessed PZ and TZ masks ensures that the dilation only fills gaps between the zones and does not extend into unrelated areas. Finally, the conjunction with the negated TZ mask guarantees that the post-processed PZ does not overlap with the unprocessed TZ. Meanwhile, the dilation of TZ mask in the function 4 fills the remaining small gaps between the zones. The following conjunction with the closing of the union of the unprocessed zones restricts the effect of dilation to the region between the zones. The final conjunction with the negated and processed PZ ensures that the processed TZ does not overlap with the processed PZ. The ball kernel of a 10-pixel radius is used for binary morphological closing operation. The shape and size of the kernels of dilation and morphological closing operations are empirically selected to fill small gaps while preserving the anatomical shape of the prostate zones.

**Figure 12.**
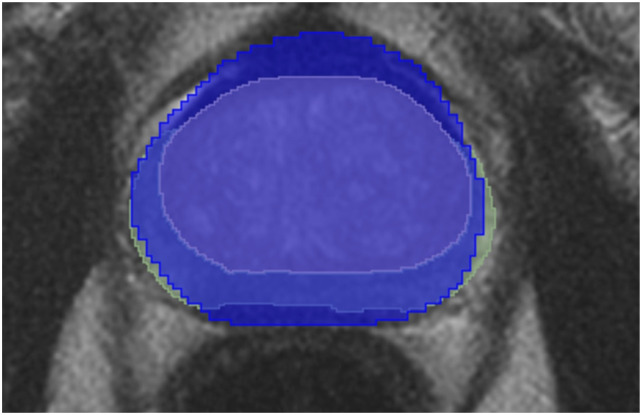
A single slice of post-processed SM4TZPZS workflow result where green area represents PZ, yellow area represents TZ and blue area represents prostate gland.

An example of post-processed prostate zones is provided in Figure 12 where the yellow area is a TZ, the green area represents a PZ, and the blue area is a prostate mask provided by NVI.

A lot of post-processed prostate zones contain regions that do not intersect the prostate mask. Therefore, all the post-processed prostate zone masks are further refined by acquiring the intersection between the prostate mask and the prostate zone mask. The refined prostate masks are used for training of the deep neural network models, while unrefined ones are used for the evaluation of ensembles and the baseline model.

Lastly, the data size is 119 cases. The data is imbalanced and cases with a cancerous region are under-represented. The distribution of cases with at least one cancerous region and cases without a cancerous region in each zone is presented in Table 5. This sample size is considered sufficient for the comparison of the proposed prostate cancer segmentation ensemble accuracies. Although the number of positive cases is limited, it is representative of the underlying population distribution and allows for reliable relative comparison.

**Table 5.** The number of cases with at least one cancerous region and number of healthy cases in each zone.

Region	Cases with cancerous regions	Cases without cancerous regions
Prostate region	42	77
TZ	34	85
PZ	41	78

### Experiment setup

As mentioned in the previous section, the dataset is imbalanced, as cases with a cancerous region are under-represented. To address this issue, the evaluation of ensembles and the baseline model consists of performing 5-fold cross-validation. Patients with cancerous regions are evenly distributed across the folds. 5-fold cross-validation is performed for each ensemble and the baseline model with the same folds. Thus, all deep neural network models are trained 5 times, each time using a different fold. Consequentially, the ensembles and the baseline model are evaluated 5 times as well, each time using a different fold. Four of the five resulting folds contain 24 patients. Therefore, for each fold models used in the ensembles are trained on 95 cases. For each fold the resulting ensembles are tested on 24 cases. The last fold consists of 23 patients. Hence, for this fold each model used in the ensembles are trained on 96 patients and each resulting ensemble is tested on 23 cases.

All deep neural network models are trained using nnU-Net method. The training data augmentation consists of random rotations, random scaling, Gaussian noise, Gaussian blur, gamma correction and mirroring. Each deep neural network model is trained using a batch size of 4 for 1000 epochs. Optimization is performed using stochastic gradient descent with Nesterov momentum (*μ* == 0.99) and an initial learning rate of 0.01. The learning rate is updated at the beginning of each epoch using polynomial decay schedule, defined in the equation (5). In this equation *lr*_0_ denotes the initial learning rate, *e* represents the current epoch, *E* represents total number of epochs and *lr*_*e*_ corresponds to the adjusted learning rate at epoch *e*. Each training is performed with a different random seed. All trainings and inference experiments are conducted on the Nvidia DGX1 GPU server equipped with 8xV100 system architecture.

(5) $$lr_{e}=lr_{0}\times {\left(1-\frac{e}{E}\right)}^{\exp}$$

Overall, 4 ensembles are evaluated and compared. They are described in Methods section as well as the baseline model, *prostate* (*All*, *Zones*). The evaluation is performed for each patient separately by calculating the DSC and the *DSC*_*τ*_. Unfortunately, if the patient does not have a cancerous region, then both *DSC*_*τ*_ and original DSC cannot be calculated. Therefore, for each ensemble and baseline model, the percentage of patients (false positive patients), for whom the result of the ensemble or the baseline model includes a cancerous region while the ground truth does not, is computed. This percentage is calculated for each fold separately as well. The dataset contains 77 patients whose ground truth does not contain a cancerous region. Hence, only these 77 patients are used for the calculation of false positive patients metric. Meanwhile, the *DSC*_*τ*_ and the original DSC are calculated for 42 patients whose ground truth contained cancerous regions.

## Results

Firstly, *DSC*_*τ*_ and original DSC, calculated from 5-fold cross-validation results, are aggregated. The aggregation consists of calculating the mean and standard deviation (Std). These metrics are presented in Table 6. The Ensemble/model column indicates the compared ensemble and the baseline model. The aggregated results indicate that Ensemble 3 achieves the most accurate results, followed closely by Ensemble 4. Furthermore, the results indicate that the proposed metric, *DSC*_*τ*_, yields higher values than the conventional DSC, reflecting reduced penalization of boundary discrepancies within the predefined tolerance.

**Table 6.** Mean and standard deviation of prostate cancer segmentation results acquired from 5-fold cross-validation. These metrics are calculated on collected metrics *DSC*_*τ*_ and DSC.

Ensemble/Mode	DSC	*DSC*_*τ*_
Ensemble 1	0.1613 +/- 0.2271	0.2116 +/- 0.3015
Ensemble 2	0.1828 +/- 0.2381	0.2512 +/- 0.3269
Ensemble 3	**0.2264 +/- 0.2611**	**0.2977 +/- 0.3443**
Ensemble 4	0.2155 +/- 0.2580	0.2829 +/- 0.3395
Baseline	0.1787 +/- 0.2387	0.2360 +/- 0.3152

The bold font indicates the highest achieved metrics.

In addition, Friedmann test results indicate a significant difference between the compared ensembles and the baseline model. The p-value when comparing *DSC*_*τ*_ is 0.003107, while the p-value of DSC comparison is 0.00067. Both of those values are lower than the significance level, 0.05 (confidence level 95%). Therefore, the hypothesis *H*_0_ is rejected, which indicates a statistically significant difference between the compared ensembles and the baseline model.

Conover post hoc test for unreplicated blocked data^41^ is performed, as Friedmann test results indicate a statistically significant difference between compared ensembles and baseline model. The p-values are adjusted by using the Benjamini/Hochberg (non-negative) method. The Conover post hoc tests are performed twice - once with the DSC metric and the other - with *DSC*_*τ*_ metric. The results of the test are presented in Table 7 where the column comparison indicates which ensembles/baseline model is compared, the columns p-value (DSC) and p-value (*DSC*_*τ*_) represent p-values of the tests when testing on DSC and *DSC*_*τ*_ respectively.

**Table 7.** Conover post hoc test results. The tests are performed on collected metrics *DSC*_*τ*_ and DSC. The metric on which the p-value is acquired is indicated in parenthesis.

Comparison	p-value (DSC)	p-value (*DSC*_*τ*_)
Ensemble 1 - Ensemble 2	0.7059	0.5352
Ensemble 1 - Ensemble 3	**0.0079**	**0.0189**
Ensemble 1 - Ensemble 4	**0.0179**	0.0656
Ensemble 1 - Baseline	0.7059	0.5758
Ensemble 2 - Ensemble 3	**0.0148**	0.0741
Ensemble 2 - Ensemble 4	**0.0462**	0.2490
Ensemble 2 - Baseline	0.6042	0.2533
Ensemble 3 - Ensemble 4	0.7059	0.5710
Ensemble 3 - Baseline	**0.0043**	**0.0069**
Ensemble 4 - Baseline	**0.0096**	**0.0189**

The bold font indicates the p-values that confirm the statistically significant difference.

Since the p-values are below the significance level, *H*_0_ is rejected, indicating that there is a statistically significant difference between the results of the baseline model *prostate* (*All*, *Zones*) and Ensembles 3, 4. Meanwhile, there is no statistically significant difference between the results of the baseline model *prostate* (*All*, *Zones*) and Ensembles 1, 2 as p-values are higher than the significance level and therefore, *H*_0_ is not rejected. The other results of the Conover post hoc test show that there is no statistically significant difference between the results of Ensembles 3 and 4 as well as the results of Ensembles 1 and 2. However, the DSC metrics of Ensembles 1, 2 and the results of Ensembles 3, 4 statistically significantly differ while *DSC*_*τ*_ metrics - do not.

**Figure 13.**
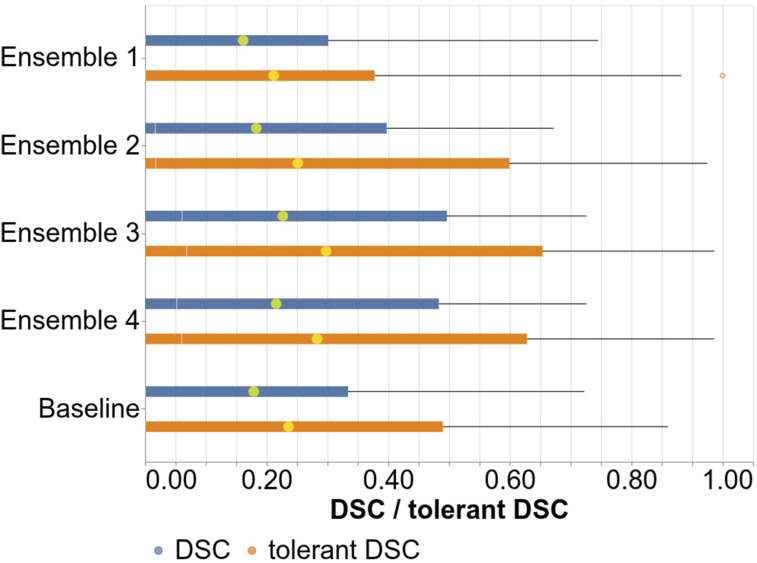
Boxplots of DSC (blue) and *DSC*_*τ*_ (orange) prostate cancer segmentation results acquired from 5-fold cross-validation.

Moreover, the *DSC*_*τ*_ and DSC, calculated from 5-fold cross-validation results, are visualized as boxplots in Figure 13. The vertical axis indicates compared ensembles and the baseline model, while the vertical one indicates aggregated DSC and *DSC*_*τ*_. The orange boxplots indicate *DSC*_*τ*_ while the blue ones indicate DSC. The yellow dots on boxplots represent the mean DSC and mean *DSC*_*τ*_ for their respective boxplots. The boxplot indicates the same conclusions as Table 6.

Furthermore, Table 8 contains the percentage of patients (false positive patients), for whom the result of the ensemble or the baseline model includes a cancerous region while the ground truth does not. Just as in Table 6, the Ensemble/model column indicates the compared ensembles and the baseline model. Meanwhile, the false positive patients column contains the false positive patients calculated for the whole dataset. This metric indicates that the baseline model, *prostate* (*All*, *Zones*), segments the prostate cancer for patients erroneously the least.

**Table 8.** The percentage of patients for whom the result of the ensemble or the baseline model includes a cancerous region while the ground truth does not (false positive patients).

Ensemble/Model	False positive patients
Ensemble 1	24.68
Ensemble 2	28.57
Ensemble 3	35.06
Ensemble 4	27.27
Baseline	**19.48**

The bold font indicates the lowest achieved false positive patients.

Table 9 contains the distributions of predicted lesions across patient by each compared ensemble and the baseline model. The Ensemble/model column lists all compared ensembles alongside the baseline model. Each column within the No of predicted lesions group reports the number of patients for whom the corresponding ensemble or the baseline model produced that specific number of predicted lesions. The distributions are similar to the true lesion count. In total, 77 patients in the dataset do not contain any lesions, 36 patients contain one lesion, and 6 patients contain two lesions. Ensemble 4 predicts no lesions for exactly 77 patients, predicts one lesion for four fewer patients than the true count, and predicts two lesions for two more patients than the true count. Ensemble 1 produced lesion count distribution is slightly less similar to true lesion count. It predicts no lesions for one additional patient, predicts one lesion for seven fewer patients, and predicts two lesions for three more patients. Meanwhile, the lesion count distributions produced by Ensemble 2 and Ensemble 3 deviate significantly from the actual lesion count. Ensemble 2 predicts no lesions for six fewer patients, whereas Ensemble 3 predicts no lesions for ten fewer patients. Ensemble 2 predicts one lesion for five fewer patients, while Ensemble 3 predicts one lesion for four fewer patients. Ensemble 2 predicts two lesions for nine additional patients, compared with eleven additional patients for Ensemble 3. The baseline model’s distribution deviates most from the true lesion counts. It predicts no lesions for 13 additional patients, one lesion for 12 fewer patients, and two lesions for 2 fewer patients. The results presented in Table 9 indicate that the baseline model tends to under detect lesions. In contrast, the compared ensembles generally predict lesion counts that are closer to the actual numbers.

**Table 9.** Distributions of predicted lesions across patients. Each distribution is expressed as the number of patients for whom the ensemble or the baseline model generated the corresponding number of lesions.

Ensemble/model	No of predicted lesions					
	0	1	2	3	4	6
Ensemble 1	78	29	9	1	1	1
Ensemble 2	71	31	15	2	0	0
Ensemble 3	67	32	17	3	0	0
Ensemble 4	77	32	8	1	1	0
Baseline	90	24	4	0	1	0

Moreover, the compared ensembles tend to predict more than two lesions per patient, even though the deep neural network models were trained on a dataset in which the maximum number of lesions per patient is two. However, this overestimation affects only two to three patients per ensemble. The baseline model overestimates even less, predicting four lesions for only a single patient.

Table 10 contains the distributions of undetected lesions across patients by each compared ensemble and the baseline model. The Ensemble/model column represents the compared ensembles and the baseline model. Each first column within the No of missed lesions group reports the number of patients for whom the corresponding ensemble or the baseline model failed to produce the lesions that overlap with that specific number of predicted lesions. Meanwhile, every second column within this group represents the percentage of such patients. These percentages are calculated based on the 42 patients who have at least one lesion. The results presented in Table 10 indicate that Ensemble 3 achieves the highest accuracy in lesion localization. Ensembles 2 and 4 achieves marginally less accurate results, while Ensemble 1 and the baseline model achieved the least accurate results when it comes to lesion localization. This finding is consistent with the results presented in Table 6.

**Table 10.** Distributions of missed lesions across patients. In total 42 patients had at least one lesion. Each distribution is expressed as the number and the percentage of patients for whom the ensemble or the baseline model missed the corresponding number of lesions.

Ensemble/Model	No of missed lesions					
	0		1		2	
Ensemble 1	16	38.10%	24	57.14%	2	4.76%
Ensemble 2	19	45.24%	21	50.00%	2	4.76%
Ensemble 3	**22**	**52.38%**	**19**	**45.24%**	1	2.38%
Ensemble 4	20	47.62%	21	50.00%	1	2.38%
Baseline	16	38.10%	25	59.52%	1	2.38%

The bold font indicates the lowest number of missed lesions.

**Figure 14.**
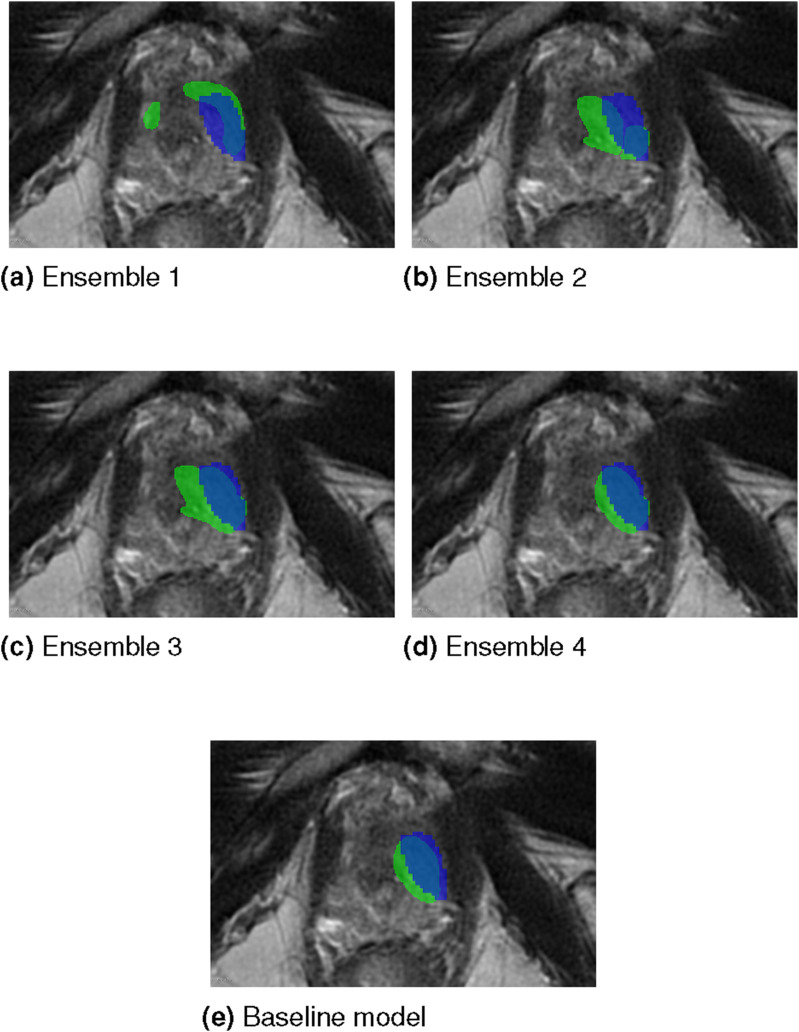
Single patient, single slice examples of segmentation results (green areas) produced by each ensemble and the baseline model, highlighting cases where the predictions overlap with the ground truth lesions (blue areas). The results are overlayed on the T2 sequence.

**Figure 15.**
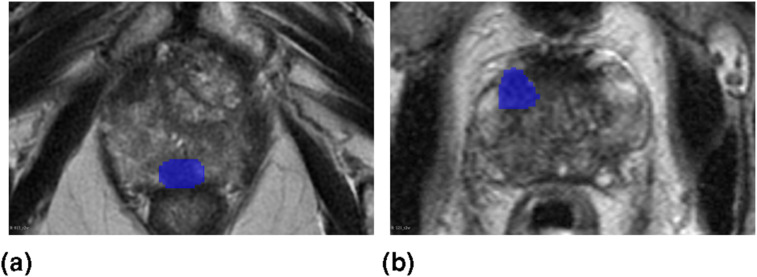
The single slice examples of patients with lesions (blue areas) that none of the ensembles as well as the baseline model localized. The results are overlayed on the T2 sequence. The examples (a) and (b) are different patients.

Figure 14 presents representative single slice segmentation results for each proposed ensemble alongside the baseline model, highlighting instances in which the predicted lesion masks overlap with the ground truth. All examples are derived from the same patient and correspond to the same anatomical slice. Green areas indicate the outputs of the respective ensemble or the baseline model, while, blue areas represent the ground truth. The results and the ground truth is overlayed on the T2 sequence. Furthermore, Figure 15 shows single slice examples of the cases for whom all of the proposed ensembles, as well as the baseline model, failed to localize the lesion. Blue areas represent the ground truth, and the ground truth is overlayed on the T2 sequence.

Two cases were observed in which the deep neural network models produced fragmented lesion predictions. In the first case, the *PZ* (*All*) model and the baseline *Prostate* (*All*, *Zones*) model segmented the same lesion into two separate components. Consequently, Ensemble 2 and the baseline model also produced a fragmented version of this ground truth lesion. In contrast, Ensemble 3 and Ensemble 4 did not exhibit this fragmentation, as the disjunction of their constituent models yielded a single, continuous lesion. The second instance of fragmentation was caused solely by the *TZ* (*All*) model. As a result, Ensemble 2 and Ensemble 3 produced a fragmented lesion in this case. However, this lesion was not localized at all by Ensemble 1, Ensemble 2, Ensemble 4 and the baseline model. Overall, no additional cases of lesion fragmentation were observed, allowing to conclude that these two instances are outliers and that the proposed ensembles generally do not fragment lesions.

**Figure 16.**
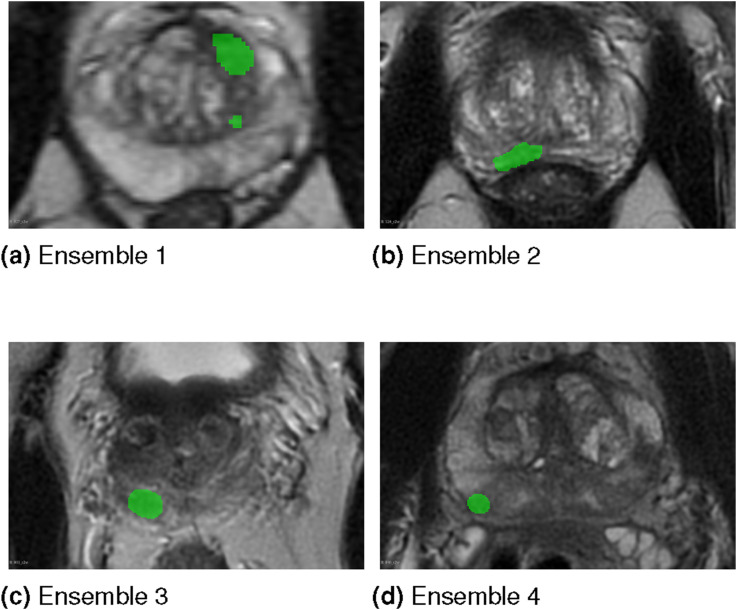
The single slice examples of generated spurious foci (green areas) by each ensemble. The results are overlayed on the T2 sequence.

Tables 11 and 12 contain the distributions of spurious foci across patients generated by the compared ensembles and the baseline model. The Ensemble/model column indicates the compared ensembles as well as the baseline model. In Table 11 each column within the No of spurious foci group reports the number of patients for whom the given ensemble or the baseline model incorrectly generated a given number of lesions which do not overlap with any ground truth lesion. Meanwhile, in Table 12 each column within the No of spurious foci group reports the percentage of patients for whom the given ensemble or the baseline model generated a given number of spurious foci. These percentages are calculated based on all 119 cases present in the dataset. The results presented in these tables indicate that the baseline model generates the least spurious foci. This observation confirms the findings made from the results presented in Table 8. Figure 16 shows the single slice example of the generated spurious foci by each proposed ensemble. Each example corresponds to a different patient for whom the baseline model did not produce any spurious foci. Green areas indicate the outputs of the respective ensemble. The results are overlayed on the T2 sequence.

**Table 11.** Distributions of spurious foci across patients generated by the compared ensembles and the baseline model. Each distribution is expressed as the number of patients exhibiting each number of generated spurious foci.

Ensemble/Model	No of spurious foci					
	0	1	2	3	4	6
Ensemble 1	94	17	5	1	1	1
Ensemble 2	86	24	9	**0**	**0**	**0**
Ensemble 3	83	26	9	1	**0**	**0**
Ensemble 4	95	20	3	**0**	1	**0**
Baseline	**108**	**10**	**0**	**0**	1	**0**

The bold font indicates the lowest number of generated spurious foci.

**Table 12.** Distributions of spurious foci across patients generated by the compared ensembles and the baseline model. Each distribution is expressed as the percentage of patients (119) exhibiting each number of generated spurious foci.

Ensemble/Model	No of spurious foci					
	0	1	2	3	4	6
Ensemble 1	78.99	14.29	4.20	0.84	0.84	0.84
Ensemble 2	72.27	20.17	7.56	**0.00**	**0.00**	**0.00**
Ensemble 3	69.75	21.85	7.56	0.84	**0.00**	**0.00**
Ensemble 4	79.83	16.81	2.52	**0.00**	0.84	**0.00**
Baseline	**90.76**	**08.40**	**0.00**	**0.00**	0.84	**0.00**

The bold font indicates the lowest percentage of generated spurious foci.

## Discussion

The result of the presented experiment evaluates possible improvements to the solution of the prostate cancer segmentation task. The accurate solution of this task is important as it can greatly assist radiologists in evaluating mpMRI for further diagnostic assessment of prostate cancer.

In this paper, four ensembles are proposed for the prostate cancer segmentation task. Moreover, these ensembles are compared with the baseline model, *prostate* (*All*, *Zones*). For the comparison, a new metric, *DSC*_*τ*_, is introduced, which is a modified DSC, *DSC*_*τ*_, and it accounts for a segmentation error in the ground truth. The comparison is performed with both the *DSC*_*τ*_ and the original DSC metric.

All of the proposed ensembles employ models trained with nnU-net method. The baseline model is trained with nnU-net method as well. Thus, each model’s architecture is equivalent. The main difference is the prostate zone within which the model segments prostate cancer. Each model can be ran in parallel as none of them depend on the output of the other. Hence, the difference between inference time of each ensemble can be negated with sufficient computational resources. However, the ensembles differ in the number of employed models. Thus, different ensemble requires larger computational resources. The largest ensemble is Ensemble 3, which employs 3 nnU-Net models. Meanwhile, the other 3 ensembles consist of only 2 models. Therefore, it is impractical to use Ensemble 3 if at least one of the other 3 ensembles achieve similar results.

Furthermore, the compared ensembles as well as the baseline model employ prostate zone masks. Therefore, the isolated quantitative effect of zonal information cannot be directly assessed within this study. However, the consistent use of zonal masks across ensembles ensures that performance differences are not confounded by unequal access to anatomical prior, allowing a more controlled evaluation of ensemble strategies. Moreover, prostate zone masks provide an explicit anatomical prior that guides the network toward region specific representations, which is particularly relevant given the known differences in cancer prevalence and imaging characteristics between prostate zones. In addition, the positive effect on prostate cancer segmentation accuracy was indicated by research presented in prior publications.

Both collected *DSC*_*τ*_ and DSC indicate that Ensemble 3 achieves the most accurate results in prostate cancer segmentation tasks. This ensemble achieves 4.77% higher mean DSC than the baseline model *prostate* (*All*, *Zones*). Furthermore, Ensemble 3 achieves 6.17% higher mean *DSC*_*τ*_ than the baseline model *prostate* (*All*, *Zones*). However, Ensemble 4 is only marginally less accurate than Ensemble 3. Ensemble 4 achieves 1.09% lower mean DSC than Ensemble 3. Moreover, Ensemble 4 achieves 1.48% lower mean *DSC*_*τ*_ and 0.84% than Ensemble 3. The result of Ensemble 3 consists of the results of the three models, while the result of Ensemble 4 is acquired from two models. Therefore, Ensemble 4 uses fewer models, and both Ensemble 3 and Ensemble 4 can be considered as the most suitable solution to the prostate cancer segmentation task. Moreover, this observation is consistent with the results of the missed lesions analysis.

Meanwhile, the baseline model *prostate* (*All*, *Zones*) is the least erroneous when segmenting prostate cancer among patients. This model achieves the lowest percentage of false positive patients. Moreover, this finding is further supported by the analysis of generated spurious foci. These results indicate that the use of disjunction within the ensembles leads to an accumulation of false positives. This is consistent with the observation that Ensemble 3, which combines three deep neural network models rather than two, yields substantially more false positive cases than the other ensembles. Alternative ensemble strategies, such as majority voting, could potentially reduce false positive rates, although this would likely come at the cost of decreased accuracy. Furthermore, the *prostate* (*All*, *Zones*) model exhibits a reduction in false positive patient cases by less than 10% compared to the next two ensembles, which achieve the lowest percentage of false positive patients, Ensemble 1 and Ensemble 4. Considering that sensitivity in medicine is more important than specificity, the difference in false positive patients between Ensemble 1, Ensemble 4 and *prostate* (*All*, *Zones*) model can be considered insignificant.

Moreover, Ensemble 1 and Ensemble 2 perform significantly worse than Ensemble 3 and Ensemble 4. Furthermore, the accuracy of Ensemble 4 is only marginally worse than Ensemble 3. All ensembles, except Ensemble 4, employ TZ specific prostate cancer segmentation deep neural network model. Moreover, both Ensemble 3 and Ensemble 4 use model which segments prostate cancer in the whole prostate gland. These findings indicate that TZ specific prostate cancer segmentation models do not improve overall performance. This observation is consistent with prior studies. Moreover, it aligns with anatomical considerations, including the difficulty of distinguishing cancerous regions in TZ from benign hyperplasia nodules, the diffuse background heterogeneity and multinodularity of TZ, and the effects of stromal asymmetry on cancer segmentation within TZ.

Furthermore, Ensemble 1 accuracy is worse than the other ensembles and the baseline model. The difference between Ensemble 1 and the other ensembles, as well as the baseline model, is the use of zone specific imaging sequences in prostate cancer segmentation models. These results indicate that employing all three mpMRI sequences in prostate cancer segmentation models is preferable to using zone specific sequences. However, Ensemble 1 achieves the lowest false positive patients among compared ensembles. This suggests that the use of all imaging sequences may increase the likelihood of generating more spurious foci.

Lastly, mean *DSC*_*τ*_ is higher than the original mean DSC. Ensemble 1 achieves 5.03% higher *DSC*_*τ*_ than the original DSC. Ensemble 2 achieves 6.84% higher *DSC*_*τ*_ than the original DSC. Ensemble 3 achieves 7.13% higher *DSC*_*τ*_ than the original DSC. Ensemble 4 achieves 6.74% higher *DSC*_*τ*_ than the original DSC. The model *prostate* (*All*, *Zones*) achieves 5.73% higher *DSC*_*τ*_ than the original DSC. This result indicates that the *DSC*_*τ*_ accounts for ground truth segmentation errors. The incremental contribution of the proposed metric lies in its ability to account for clinically acceptable boundary deviations that are penalized by the conventional DSC. In prostate cancer segmentation, small spatial discrepancies are common due to inter observer variability, yet these deviations often have minimal clinical impact. The conventional DSC treats all mismatches equally, regardless of their spatial magnitude. In contrast, the proposed *DSC*_*τ*_ introduces a tolerance margin that considers small boundary offsets as acceptable matches. This allows the metric to distinguish between clinically negligible boundary deviations and truly significant segmentation errors. Although the ranking of the evaluated methods remains similar in our experiments, the *DSC*_*τ*_ provides a more clinically meaningful interpretation of segmentation quality by reducing the impact of minor boundary inconsistencies. This is particularly relevant for prostate cancer segmentation, where small contour variations frequently occur even among expert annotations. Therefore, the proposed metric does not aim to replace the conventional DSC but rather to complement it by providing an evaluation that better reflects clinically acceptable segmentation accuracy.

Overall, considering that Ensemble 4 is the only ensemble that achieved competitive results when comparing *DSC*_*τ*_, *DSC* and false positive patients in the experiment presented in this paper, this research concludes that Ensemble 4 is the most suitable solution to the prostate cancer segmentation task. The achieved metrics are comparable to those reported in previous studies on prostate cancer segmentation, such as the work presented in Ref. 14. However, both the baseline model and the proposed ensembles exhibited high false positive rates and low DSC, indicating that neither approach currently provides a sufficiently accurate solution for prostate cancer segmentation. These limitations are likely influenced by the relatively small dataset size, which not only restricts the learned lesion characteristics but also introduces a bias toward smaller lesions, as reflected in the lesion size distribution. Consequently, the ensembles may have been predominantly trained on features associated with smaller lesions, potentially impairing their ability to accurately segment larger ones. While the precise impact of this imbalance is difficult to assess given the limited sample size, evaluating the proposed ensembles on substantially larger and more representative datasets is a critical direction for future work. Such evaluation could help mitigate false positives, clarify the effect of lesion size distribution, and better assess robustness across a wider range of lesion sizes. Moreover, if low DSC values and high false positive rates persist on larger datasets, the proposed ensembles may still be valuable for prostate cancer localization, in which case alternative performance metrics, such as the area under the receiver operating characteristic curve or average precision, may be more appropriate than DSC, as DSC penalizes small boundary discrepancies. Furthermore, prior studies on prostate cancer segmentation that utilized larger datasets, such as,^18,19^ reported notably higher performance metrics. Lastly, it should be emphasized that the primary objective of this study is the empirical comparison of the proposed ensembles with the baseline model, rather than the development of a clinically deployable prostate cancer segmentation method. The findings highlight the most feasible solution among the compared approaches for the prostate cancer segmentation task. Nevertheless, further validation on emerging large scale publicly available datasets is necessary.

The dataset was collected from 11 different institutions, which supports the generalizability of the findings across institutional settings. In addition, the data were acquired using a wide range of mpMRI scanners and imaging parameters, further enhancing the applicability of the results to heterogeneous clinical environments. However, the substantial variability in acquisition protocols may have negatively affected the accuracy of the evaluated ensemble. This limitation could be mitigated in future work by training and evaluating the models on a larger dataset.

The results obtained in the experiment of this paper indicate one further possible future research direction on the prostate cancer segmentation task, the incorporation of another mpMRI sequence, Dynamic Contrast Enhancement, into the result of the ensembles described in this paper.

## Conclusion

This study demonstrates that the proposed ensembles incorporating prostate zone masks can significantly improve the accuracy of prostate cancer segmentation. In contrast, the use of zone specific mpMRI sequences did not yield additional performance gains. The highest segmentation accuracy was achieved by the ensemble combining two deep neural network models: one trained to segment cancerous regions across the entire prostate and another focused specifically on the peripheral zone. Despite these promising results, the experiments were conducted on a dataset of limited size, which negatively affect the accuracy of the compared ensembles. Consequently, further validation on larger datasets is required to confirm the robustness of the proposed approach. Finally, the results indicate that the proposed tolerant Dice similarity coefficient effectively accounts for high inter observer variability, highlighting its suitability as an evaluation metric for prostate cancer segmentation tasks.

## Data Availability

The data are not publicly available due to restrictions of the bioethical agreement (number 2020/5-1229-714).*
